# CsmA Protein is Associated with BChl *a* in the Baseplate Subantenna of Chlorosomes of the Photosynthetic Green Filamentous Bacterium *Oscillochloris trichoides* belonging to the Family *Oscillochloridaceae*

**DOI:** 10.1155/2011/860382

**Published:** 2011-09-15

**Authors:** Anastasiya Zobova, Alexandra Taisova, Eugeny Lukashev, Nataliya Fedorova, Ludmila Baratova, Zoya Fetisova

**Affiliations:** ^1^A.N. Belozersky Institute of Physico-Chemical Biology, Building A, M.V. Lomonosov Moscow State University, Moscow 119991, Russia; ^2^Department of Biophysics, M.V. Lomonosov Moscow State University, Moscow 119991, Russia

## Abstract

The baseplate subantenna in chlorosomes of green anoxygenic photosynthetic bacteria, belonging to the families *Chloroflexaceae* and *Chlorobiaceae*, is known to represent a complex of bacteriochlorophyll (BChl) *a* with the ~6 kDa CsmA proteins. Earlier, we showed the existence of a similar BChl *a* subantenna in chlorosomes of the photosynthetic green bacterium *Oscillochloris trichoides*, member of *Oscillochloridaceae*, the third family of green photosynthetic bacteria. However, this BChl *a* subantenna was not visually identified in absorption spectra of isolated *Osc. trichoides* chlorosomes in contrast to those of *Chloroflexaceae* and *Chlorobiaceae*. In this work, using room and low-temperature absorbance and fluorescence spectroscopy and sodium dodecyl sulfate polyacrylamide gel electrophoresis analysis of alkaline-treated and untreated chlorosomes of *Osc. trichoides*, we showed that the baseplate BChl *a* subantenna does exist in *Oscillochloridaceae* chlorosomes as a complex of BChl *a* with the 5.7 kDa CsmA protein. The present results support the idea that the baseplate subantenna, representing a complex of BChl *a* with a ~6 kDa CsmA protein, is a universal interface between the BChl *c* subantenna of chlorosomes and the nearest light-harvesting BChl *a* subantenna in all three known families of green anoxygenic photosynthetic bacteria.

## 1. Introduction

Green anoxygenic bacteria comprise three phylogenetically unrelated families of photosynthetic bacteria: green sulfur bacteria (family *Chlorobiaceae*) and green filamentous bacteria (families *Chloroflexaceae* and *Oscillochloridaceae*) [1–3]. In 2000, the genus *Oscillochloris *was excluded from the family *Chloroflexaceae*, and a new family *Oscillochloridaceae* was proposed based on phylogenetic data and unique physiological, biochemical, and chemotaxonomical properties [3]. The photosynthetic apparatus of green anoxygenic bacteria has a particular molecular organization and contains chlorosomes, unique extramembrane light-harvesting antennae structures [4, 5]. The group of chlorosome-containing bacteria was enlarged by the recently discovered new phototrophic chlorosome-containing organism *Candidatus *Chloracidobacterium thermophilum from the phylum *Acidobacteria* [6], and it is a surprising fact. 

Chlorosomes are ellipsoid oblong bodies of about 70–260 nm long, 30–100 nm wide, and 10–30 nm thick (depending on the species) attached to the inner surface of the cytoplasmic membrane. They are enveloped by a protein-lipid monolayer of 2-3 nm width [7]. The bulk of light-harvesting pigments (including various types of bacteriochlorophylls (BChl) *c *and/or *d *or *e *and carotenoids) is located within chlorosomes. The organization of the major BChl *c/d/e* in chlorosomes is based upon pigment-pigment interactions and not upon pigment-protein interactions as in other photosynthetic antenna systems [8, 9]. These BChl *c/d/e* oligomers form either rod- (with a diameter of 5–10 nm) [10–13] or lamellar-like structures [14–16], arranged parallel to the longer chlorosome axis. Recently, using computational integration of two different bioimaging techniques, solidstate NMR and cryoEM, it was concluded that pairs of alternating *syn*-*anti*-ligated BChl* c* and *d *stacks form concentric helical nanotubes in chlorosomes from a *Cba. tepidum *mutant [17].

A minor amount of BChl *a* is also present in the chlorosome [18, 19]. This BChl *a* is located in the baseplate, observed in freeze-fracture electron-micrographs of chlorosomes from *Chloroflexaceae * and *Chlorobiaceae* species as a 5-6 nm thick paracrystalline layer [10, 11]. It should be noted that the baseplate thickness was 3 nm according to recent cryo-electron tomography of *Cfx. aurantiacus *chlorosomes [20]. The baseplate is believed to be a pigment-protein complex that is located at the base of the chlorosome [4]. The B798 light-harvesting baseplate of the chlorosome antenna complex of *Chloroflexus aurantiacus *was isolated and characterized. The isolated baseplate consists of BChl *a*, *β*-carotene, and the 5.7 kDa CsmA protein [21]. The baseplate is in contact with the cellular cytoplasmic membrane and mediates excitation energy transfer to reaction centers located in the cytoplasmic membrane [22]. In green sulfur, bacteria an additional layer (not found in *Chloroflexus*) composed of a water-soluble BChl* a*-protein, the Fenna-Matthews-Olson (FMO) protein, is situated between the baseplate and the cytoplasmic membrane [5, 23].

The chlorosome envelope consists of monogalactosyl diglyceride and several polypeptides [4]. Chlorosomes from the green filamentous bacterium *Cfx. aurantiacus *have been reported to contain three major proteins, CsmA, CsmM, and CsmN (which are the products of the *csmA*,* csmM, *and *csmN* genes, resp.), with molecular masses 5.7, 11, and 18 kDa [24–26]. Additional minor proteins, 6 kDa protein, CsmO (9,5 kDa), CsmY (22 kDa), and CsmP (20 kDa), were also observed [26, 27]. CsmA is the smallest and most abundant of these proteins. 

In *Chl. tepidum*, ten chlorosome proteins have been identified: CsmA (6,2 kDa), CsmB (7,5 kDa), CsmC (14,3 kDa), CsmD (11,1 kDa), CsmE (6,7 kDa), CsmF (7,6 kDa), CsmH (21,6 kDa), CsmI (25,9 kDa), CsmJ (21,8 kDa), and CsmX (24,0 kDa), all of which are located in the protein-lipid envelope of the chlorosome [19, 28]. The 6.2-kDa CsmA accounts for about half of the protein present in the chlorosome [19]. Recently, the first high-resolution structural model of CsmA from *Chl. tepidum* has been presented [29]. Besides, gel electrophoresis and immunoblotting experiments have shown that the same set of ten chlorosome proteins are present in *Chl. tepidum*, *Chl. vibrioforme*, and *Chl.phaeobacteroides *[30, 31]. 

Strong evidences has been obtained that only CsmA is absolutely necessary for the normal assembly and organization of BChl *c *and BChl *a* within the chlorosome. All functional genes responsible for each of the nine other chlorosome proteins could be eliminated with a little phenotypic effect [32]. Besides, CsmA is a BChl *a*-binding protein in both *Cfx. aurantiacus *[21, 26] and *Chl. tepidum *[33]. CsmA binds one BChl *a *molecule and one or two carotenoid molecules per monomer [21, 26, 33] and probably forms an oligomeric, paracrystalline CsmA–BChl *a *complex [33, 34]. 

The precise function of the *Chl. tepidum* chlorosome proteins is yet under investigations. But it is obvious that each protein found in the chlorosomes of *Cfx. aurantiacus *is clearly related in sequence to a protein found in the chlorosome envelopes of the green sulfur bacteria [19, 27].

At present, it is not known how the BChl *a* subantenna in the chlorosomes from the green anoxygenic mesophilic filamentous bacteria from the recently discovered family *Oscillochloridaceae* [3] is organized. Our previous results indicated that unlike *Chloroflexaceae* species, the photosynthetic apparatus of *Oscillochloridaceae* characterized by a very large size of BChl *c* chlorosomal antenna similar to that in *Chlorobiaceae* species, so that the absorption of BChl *c* practically completely shields the absorption of other light-harvesting pigments in the near-infrared region of the absorption spectra in intact cells [35, 36]. The absorption spectra of isolated chlorosomes *Oscillochloridaceae* exhibited no BChl *a* component found in isolated chlorosomes from two other families of green bacteria, *Chloroflexaceae *and *Chlorobiaceae *[35, 37]. However, fluorescence spectra of chlorosomes and absorption spectra of acetone-methanol extracts of isolated chlorosomes from *Osc. trichoides* revealed the presence of very small amounts of BChl *a* in chlorosome samples [35, 38]. This allowed us to propose the existence of an intermediate-energy subantenna to interface the chlorosomal BChl *c* and the membrane BChl *a* ones. Nevertheless, neither use of BChl *c* synthesis inhibitors nor cultivation of this culture at high light intensity allowed us to identify visually some additional subantenna in absorption spectra of *Oscillochloridaceae* chlorosomes [36]. At the same time, the biological expedience of such intermediate-energy subantenna in the light-harvesting system of this family was shown by us theoretically [39]. 

In this work, the idea of association of BChl *a* with protein in chlorosomes of *Osc. trichoides* was probed by room- and low-temperature absorbance and fluorescence spectroscopy and sodium dodecyl sulfate polyacrylamide gel electrophoresis (SDS-PAGE) analysis of alkaline-treated and untreated chlorosomes. We showed that the baseplate BChl *a* subantenna does exist in *Oscillochloridaceae * chlorosomes as a complex of BChl *a* with the 5.7 kDa CsmA protein.

## 2. Materials and Methods

### 2.1. Growth

*Osc. trichoides *DG-6, the type strain of the species* Osc. trichoides *(327 KM MGU), was grown, as described earlier, in batch cultures with stirring under anaerobic conditions at 30°C on a modified DGN medium at a moderate light intensity (50 *μ*E m^−2^ s^−1^) from incandescent lamps [35]. 

Cells of filamentous thermophilic green bacterium *Chloroflexus aurantiacus *strain Ok-70-fl (collection belonging to Leiden University, The Netherlands) were cultivated anaerobically in batch cultures with stirring at 55°C on a standart medium [35] at light intensity 50 *μ*E m^−2^ s^−1^.

### 2.2. Electron Microscopy

The electron microscopic observations were made with a Hitachi-12 (Japan), operating at 75 kV. For ultrathin sectioning, *Oscillochloris *cells were fixed for 30 min in the culture medium at 30°C by adding 25% glutaraldehyde to a final concentration of 1% and then at room temperature for 60 min. The samples were postfixed with 1% OsO_4_ for 90 min, embedded in Epon-812, and ultrathin-sectioned by standard methods [10].

For negative staining *Osc. trichoides *chlorosomes were dialyzed against 10 mM Tris-HCl-buffer (pH 8.0) to remove sucrose, were fixed by glutaraldehyde at a final concentration of 0.1%, and, after that, were negatively stained on formvar-coated copper grids with 2% uranyl acetate.

### 2.3. Chlorosome Isolation

Chlorosomes were isolated from *Osc. trichoides* cells in a twofold successive continuous sucrose gradient (55%–20% and 45%–15%) in the presence of 10 mM sodium ascorbate and 2 M sodium thiocyanate as described earlier [35, 37].

### 2.4. Steady State Absorbance and Fluorescence Spectroscopy

Absorption spectra were recorded at room and 77 K temperature with a Hitachi-557 spectrophotometer (Japan). Glycerol was added (60% v/v) to the samples for measurements at 77 K to obtain clear samples upon cooling.

Fluorescence excitation and fluorescence emission spectra at both room temperature and 77 K were recorded using a Hitachi-850 spectrometer. Samples were prepared by diluting a sample in 50 mM Tris buffer (pH 8.0) to obtain an optical density 0.2 at the chlorosome peak 750 nm. 

Before fluorescence measurements, the chlorosomes were incubated 60 min with 20 mM sodium dithionite at 4°C to ensure strongly reduced conditions (up to −400 mV). Some samples did not contain any reducing agent (aerobic conditions). Glycerol was added (60% v/v) to the samples for measurements at 77 K. The fluorescence emission spectra recorded at excitation wavelength 720 nm. The fluorescence excitation spectra recorded at emission wavelength 820 nm.

### 2.5. Pigment Analysis

Quantitative BChl *a *and BChl *c *contents were determined according to the method developed by [40]. Samples were extracted for 20 min in the dark at 4°C with a 25-fold volume of an acetone-methanol mixture (7 : 2, v/v). The absorbance of the clarified supernatant was measured at 769 nm for BChl *a *and 663 nm for BChl *c *with a Hitachi-557 spectrophotometer. Calculations were based on molar extinction coefficients, *ε*, of 68.6 and 74 mM ^−1^ cm^−1^ for BChl *a * and BChl *c*, respectively.

### 2.6. Alkaline Treatment

Chlorosomes were treated with alkali [41] by adding 0.1 volume of 10 M NaOH to a chlorosome suspension in a 10 mM potassium phosphate buffer, pH 7.0 (final A750 was 1), and incubating the suspension at 40°C for 30 min (final pH was 12.7). After incubation, two volumes of a 1.0 M potassium phosphate buffer, pH 6.0, were added to obtain a final pH of 7.2. The suspension was further diluted with the 10 mM potassium phosphate bufer and the chlorosomes were pelleted by centrifugation (180000 g for 90 min at 4°C). The chlorosomes were washed twice with the 50 mM Tris-HCl bufer (pH 8.0), resuspended in the same buffer, and stored at −70°C.

### 2.7. SDS-PAGE

Chlorosome samples were extracted with 1.4 mL of acetone at −20°C overnight. Proteins were collected by centrifugation and dissolved in sample buffer (50 mM Tris-HCl (pH 8.6), 24% (v/v) glycerol, 8% (w/v) SDS, 2% (v/v) 2-mercaptoethanol, and 0.1% (w/v) bromophenol blue). The samples were boiled for 1 min before being loaded onto gels containing 16.5%, 10%, and 4% acrylamide as separating, spacer, and stacking gel, respectively, as described [42]. After electrophoresis, the gels were stained with Coomassie brilliant blue R-250 (CBB) or with CBB and silver.

## 3. Results and Discussion

### 3.1. Electron Microscopy

**Figure 1:**
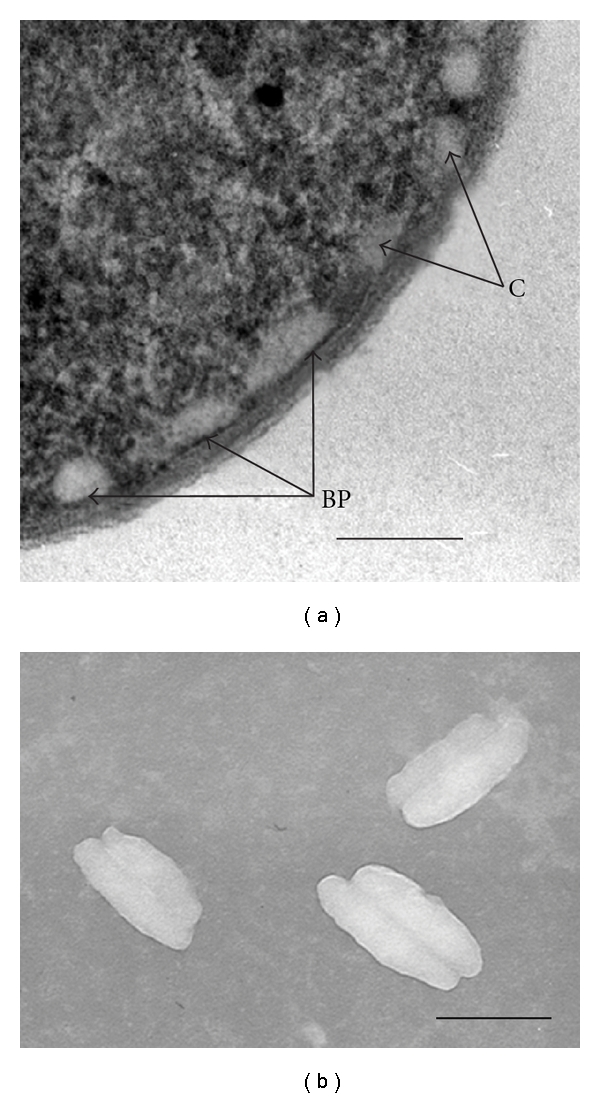
(a) Electron micrograph of ultrathin sections of *Osc. trichoides* cells. Symbols: C-chlorosome, BP-baseplate. (b) Electron micrograph of isolated chlorosomes negatively stained with 2% uranyl acetate. The bar represents 100 nm.

Micrographs of ultrathin-sectioned cells of *Osc. trichoides* clearly showed an electron-dense area (3.5–5.0 nm thick) between the chlorosome and membrane which could be interpreted to be the baseplate that anchors the chlorosome to the membrane (Figure 1(a)).

On the micrographs of chlorosomes negatively stained with 2% uranyl acetate chlorosomes appear to be cross-linked bodies probably as a result of the interactions between their baseplates possessing the hydrophobic nature (Figure 1(b)).

These data clearly show that each chlorosome consists of two spatially separate compartments pressed to each other.

### 3.2. Absorption Spectra of *Osc. trichoides* Chlorosomes

**Figure 2:**
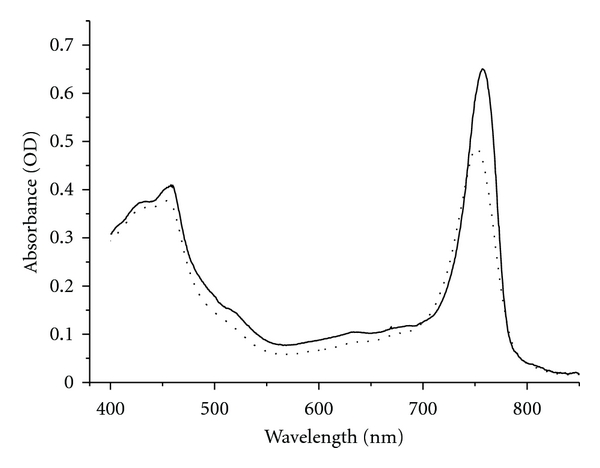
Absorption spectra of *Osc. trichoides* chlorosomes at room (dotted line) temperature and 77 K (solid line).

Earlier, it was shown that the purified chlorosomes of *Osc. trichoides* exhibited a single peak of BChl *c *at 750 nm in the near-infrared region of the absorption spectra at room temperature [35]. At 77 K, the 750-nm peak of *Osc. trichoides* was red-shifted to 758 nm and was sharper and more narrow (FWHM ≈ 35 nm) than the room temperature peak (FWHM ≈ 47 nm) (Figure 2, solid line). It should be noted that the absorbance spectrum of *Osc. trichoides* chlorosomes at 77 K showed, in addition to the near-infrared absorption band at 758 nm due to bacteriochlorophyll *c*, a weak shoulder near 805 nm, which may be attributed to BChl *a*. The presence of BChl *a *in chlorosomes was clearly visualized only by fluorescence spectroscopy measured at 77 K or by pigment extraction [35–38]. The main light-harvesting pigment in the chlorosomes was identified as BChl *c* and the minor pigment as BChl *a* [35, 37]. These results suggest there was no possibility to observe changes in the BChl *a *content in *Osc. trichoides *chlorosomes by measuring absorption spectra of chlorosomes at room and 77 K temperature (Figure 2). Actually, there were over 100 BChl *c *molecules per one BChl *a *molecule in *Osc. trichoides* chlorosomes [35–37].

**Figure 3:**
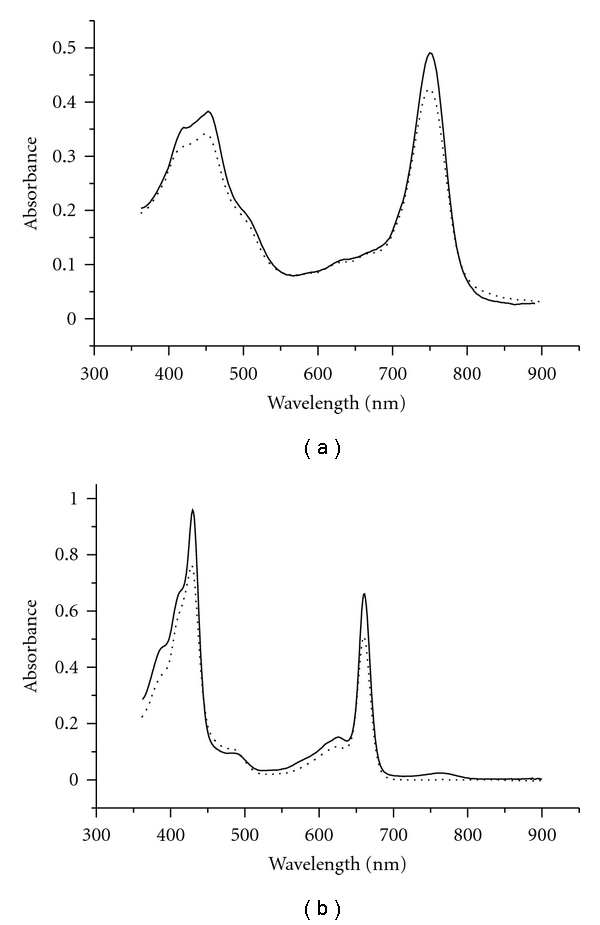
Absorption spectra of untreated (solid line) and alkaline-treated (dotted line) *Osc. trichoides* chlorosomes. (a) in 50 mM tris-buffer (pH 8,0); (b) in acetone–methanol (7 : 2).

To degrade selectively the baseplate BChl *a *in *Osc. trichoides* chlorosomes, we applied the method of alkaline treatment [41].  Figure 3(a) (dotted line) shows the effect of alkaline treatment on the absorption spectrum of the *Osc. trichoides* chlorosomes. Obviously, the absorption bands of BChl *c*, the main light-harvesting pigment in *Osc. trichoides* chlorosomes, were practically not affected by alkaline treatment.

### 3.3. Steady-State Fluorescence Excitation of *Osc. trichoides* Chlorosomes

**Figure 4:**
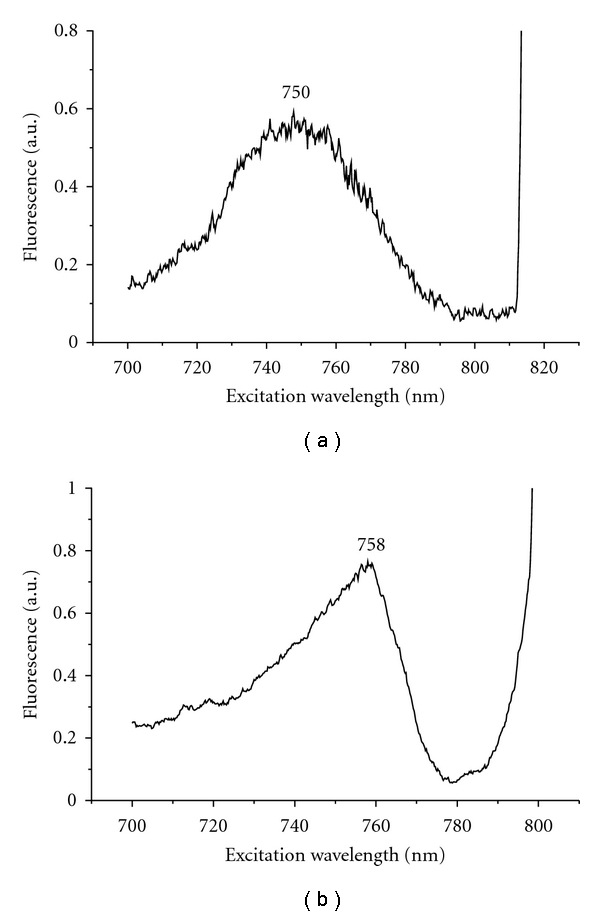
Fluorescence excitation spectra of *Osc. trichoides *chlorosomes at room temperature (a) and 77 K (b). Emission at 820 nm.

Fluorescence excitation spectra of *Osc. trichoides *chlorosomes, measured at room temperature and 77 K, are shown in Figure 4.

At room temperature, fluorescence excitation spectra of BChl *a* (Figure 4(a)) resembles the absorbance spectrum of BChl *c* (FWHM ≈ 47 nm) (Figure 2, dotted line), and positions of the maxima in both spectra are identical (750 nm). 

At 77 K, fluorescence excitation spectra of BChl *a* (Figure 4(b)) also resembles the absorbance spectrum of BChl *c* (FWHM ≈ 35 nm) (Figure 2, solid line), and positions of the maxima in both spectra are identical (758 nm).

### 3.4. Steady-State Fluorescence of Alkaline-Treated and Untreated *Osc. trichoides* Chlorosomes

**Figure 5:**
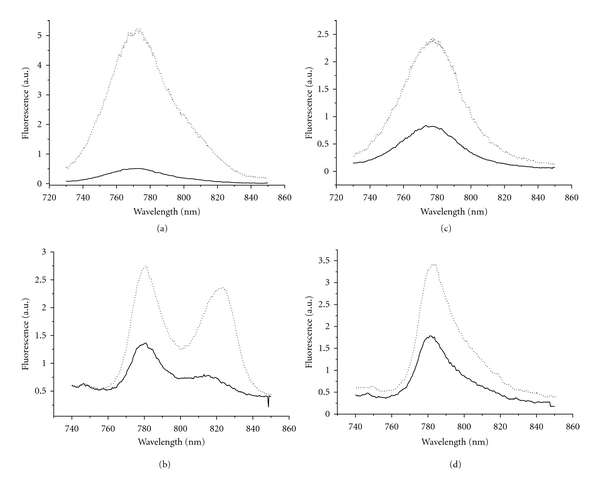
Fluorescence emission spectra of untreated and alkaline-treated *Osc. trichoides* chlorosomes at room temperature and 77 K under aerobic (solid line) and reducing (dotted line) conditions: untreated chlorosomes at room temperature (a) and 77 K (b); alkaline-treated chlorosomes at room temperature (c) and 77 K (d). Excitation at 720 nm.

As it was shown earlier, BChl *a *emission (in contrast to absorbance) could be discerned in the fluorescence emission spectra of *Osc. trichoides* chlorosomes at 77 K but not at room temperature [35, 37]. The fluorescence emission spectra of the isolated chlorosomes, when measured at 77 K, showed mainly a broad band at 780 nm, due to BChl *c*, together with another band near 820 nm, due to BChl *a*, suggesting that a baseplate is probably associated with the chlorosome (Figure 5(b)). Additionally, it was shown by us that the light-harvesting *Osc. trichoides* chlorosome antenna exhibited a highly redox-dependent BChl c fluorescence similar to Chlorobiaceae species [35–37]. For this reason, fluorescence emission spectra of untreated and alkaline-treated chlorosomes were measured at room temperature and 77 K under both aerobic and reducing conditions (dithionite-20 mM) after excitation in the *Q*_*y*_-band of BChl c at 720 nm. 

Untreated chlorosomes from *Osc. trichoides* showed different response on redox conditions at room temperature and 77 K. At room temperature, the BChl *c* emission intensity was about tenfold higher under reducing conditions than that under aerobic conditions (Figure 5(a)), while at 77 K, the intensity of the BChl *c* and BChl *a* emission increased only two- and threefold, respectively, under reducing conditions in comparison with aerobic conditions (Figure 5(b)).

Alkaline treatment had some (but also different) influence on the intensity of BChl *c* fluorescence emission both at room temperature and 77 K in *Osc. trichoides* chlorosomes (Figures 5(c) and 5(d)). At room temperature, alkaline treatment increased slightly (1,6-fold) the BChl *c* emission under aerobic conditions but decreased twofold the BChl *c *emission under reducing conditions (Figure 5(c)), as compared to untreated chlorosomes (Figure 5(a)). At 77 K, alkaline treatment increased slightly (1,3-1,4-fold) the BChl *c* emission both under aerobic and reducing conditions (Figure 5(d)). Fluorescence from BChl *a* could no longer be seen in alkaline-treated chlorosomes from *Osc. trichoides* under either reducing or aerobic conditions (Figures 5(c) and 5(d)). 

Thus, in both aerobic and reducing medium, alkaline treatment strongly decreases steady-state fluorescence intensity in the 820 nm spectral region. It is obvious that the disappearance of BChl *a* emission is caused by the removal or destruction of BChl* a* in the baseplate. At room temperature in *Osc. trichoides* alkaline-treated chlorosomes under aerobic conditions, the fluorescence intensity of BChl *c* increases only slightly and decreases about threefold on going from aerobic to reducing conditions (Figure 5(c)) in comparison with untreated chlorosomes (Figure 5(a)). At 77 K, changes in BChl *c *fluorescence intensity under different redox conditions were identical in untreated (Figure 5(b)) and alkaline-treated chlorosomes (Figure 5(d)). 

**Figure 6:**
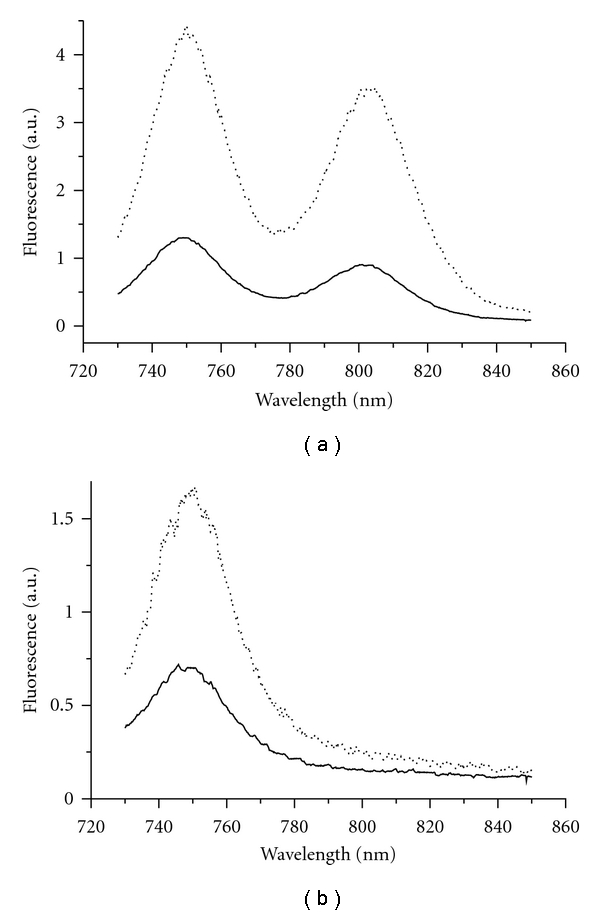
Fluorescence emission spectra of untreated (a) and alkaline-treated (b) *Cfx. aurantiacus *chlorosomes at room temperature under aerobic (solid line) and reducing (dotted line) conditions. Excitation at 720 nm.

Figure 6(a) demonstrates that under reducing conditions at room temperature, untreated chlorosomes from green filamentous bacterium *Cfx. aurantiacus* exhibited the BChl *c* emission intensity more than threefold higher than that under aerobic conditions. Alkaline treatment resulted in approximately 2-3-fold reduction of the BChl *c* fluorescence intensity under both aerobic and reducing conditions (Figure 6(b)).

Thus, our results on BChl *c* fluorescence intensity in alkaline-treated *Osc. trichoides* and *Cfx. aurantiacus* chlorosomes are very much alike: depletion of BChl *a *by alkaline treatment led to small or moderate effects on BChl *c* fluorescence intensity. 

So, we conclude that alkaline treatment of *Osc. trichoides* chlorosomes led to a selective degradation of BChl *a* in the baseplate and caused dramatic changes in the fluorescence spectra of chlorosomes while leaving BChl *c* in a form that is spectrally indistinguishable from that in untreated chlorosomes. These results are in agreement with conventional ideas about the organization of chlorosome pigments: the BChl *c* and BChl *a* pigments housed within two different (but neighboring) substructures. Selective degradation of BChl *a* would be expected if it is located outside of the chlorosomal BChl* c* body in the contact with cytoplasmic membrane, whereas BChl* c* is organized in rod or lamellar aggregates within the BChl *c* body of chlorosomes.

### 3.5. Pigment Determination in Alkaline-Treated and Untreated *Osc. trichoides* Chlorosomes

The relative contents of BChl *c *and BChl *a *pigments were determined in acetone–methanol extracts of *Osc. trichoides *chlorosomes. The absorption spectra of acetone-methanol extracts of untreated and alkaline-treated *Osc. trichoides* chlorosomes are shown in Figure 3(b). In the absorption spectra of untreated chlorosomes, two bands, at 663 nm (corresponds to monomeric BChl *c*) and 769 nm (corresponds to monomeric BChl *a*), were resolved in contrast to spectra of alkaline-treated chlorosomes that showed a single peak at 663 nm in the near-infrared region. These data confirm BChl* a* removal from *Osc. trichoides* chlorosomes upon alkaline treatment.

### 3.6. The Protein Profiles of Alkaline-Treated and Untreated *Osc. trichoides* Chlorosomes Analyzed by SDS-PAGE

**Figure 7:**
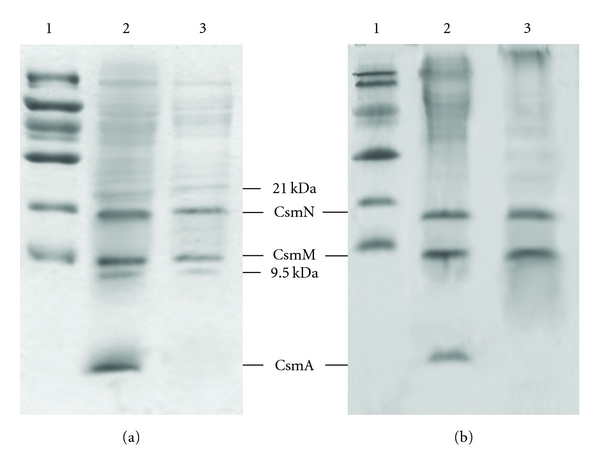
CBB-stained SDS-PAGE of untreated and alkaline-treated *Osc. trichoides* (a) and *Cfx. aurantiacus * chlorosomes (b). (a) Lane 1, molecular markers. Lane 2, untreated *Osc. trichoides* chlorosomes; Lane 3, alkaline-treated *Osc. trichoides* chlorosomes; (b) Lane 1, molecular markers. Lane 2, untreated *Cfx. aurantiacus * chlorosomes; Lane 3, alkaline-treated *Cfx. aurantiacus * chlorosomes. All *Cfx. aurantiacus *samples were adjusted to contain 4,5 *μ*g BChl *c*, while *Osc. trichoides *samples were adjusted to contain 18 *μ*g BChl *c*.

The effects of alkaline treatment on *Osc. trichoides *chlorosomal proteins were analyzed by SDS-PAGE. Five proteins (three major and two minor) were detected in untreated *Osc. trichoides* chlorosomes (Figure 7(a), lane 2). It was seen that untreated *Osc. trichoides* chlorosomes showed two major bands with molecular masses around 11 and 18 kDa and a strong broad band 5.7 kDa (Figure 7(a), lane 2). Two minor proteins with masses of 9.5 and 21 kDa were also observed. These five proteins could be visualized by CBB (Figure 7(a), lane 2) and silver staining (data not shown). Figure 7(a) (lane 3) demonstrates that alkaline treatment selectively removed 5.7 kDa protein, while the other four proteins remained largely unaffected. 

Study of protein composition of *Osc. trichoides* chlorosomes were carried out in comparison with *Cfx.aurantiacus* chlorosomes. The protein composition of native *Cfx. aurantiacus* chlorosomes and its changes after alkaline treatment are shown in Figure 7(b). Untreated *Cfx. aurantiacus* chlorosomes (lane 2) contain three major proteins with molecular masses 5.7; 11 and 18 kDa, according to literature data, designated as CsmA, CsmM, and CsmN proteins, respectively [24–26]. Figure 7(b) (lane 3) shows that alkaline treatment of *Cfx.aurantiacus* chlorosomes resulted in loss of CsmA. Obviously, protein profiles of untreated and alkaline-treated *Osc. trichoides* and *Cfx. aurantiacus* chlorosomes were very much alike. Besides, mesophile *Osc. trichoides* (family *Oscillochloridaceae*) like thermophile *Cfx.aurantiacus* (*family Chloroflexaceae*) is morphologically filamentous, shows gliding motility, and does not contain FMO protein between the chlorosome baseplate and cytoplasmic membrane of the cell. In view of this, we designated the proteins of *Osc. trichoides* chlorosomes similarly to the proteins of *Cfx. aurantiacus* chlorosomes: CsmA (5.7 kDa), CsmM (11 kDa ), and CsmN (18 kDa ). It should be noted that the amino acid sequence of the ~6-kDa band from “*Ca*. Chlorothrix halophila” (it is related phylogenetically to the *Chloroflexi *group) showed that this protein exhibits substantial sequence similarity (65% identity) to the CsmA protein from *Cfx. aurantiacus* [43]. It was shown that the photosynthetic apparatus of “*Ca*. Chlorothrix halophila” similarly to *Oscillochloris trichoides* has a combination of features that are present in both phyla of green bacteria: the chlorosome peak is similar to that of *Chlorobi* species, a minimal amount of BChl *a *is present, and the likely antenna complex is comparable to the B808-866 antenna in *Cfx. aurantiacus*.

As we reported above, alkaline treatment of *Osc. trichoides* chlorosomes led to a selective removal of BChl *a* in the baseplate. In this section, we demonstrated that alkaline treatment selectively removed CsmA protein from *Osc. trichoides* chlorosomes. Thus, a strict correlation between removal of CsmA protein and removal of BChl *a* in the baseplate was demonstrated: only this protein was removed from chlorosomes concurrently (Figure 7(a)) with the disappearance of BChl *a* fluorescence, leaving BChl *c* unchanged spectrally (Figures 5(c) and 5(d)). Selective BChl *a* and 5.7 kDa protein disappearance should be expected only in case when both of them are located out of the BChl *c* body, that is, within the baseplate of the chlorosome.

Thus, the complex study of the structure-function correlations of BChl *a* and CsmA protein in *Osc. trichoides* chlorosomes led us to the following conclusions. 

(1) The presence of BChl *a* in *Osc. trichoides* chlorosomes was confirmed (a) by the presence of the band peaking at 820 nm in the fluorescence spectrum of isolated chlorosomes at 77 K (Figure 5(b)); at room temperature, the corresponding band looks like a shoulder at 805 nm (Figure 5(a)); (b) by the presence of the band peaking at 769 nm in the absorption spectrum of acetone-methanol extract of chlorosome pigments at room temperature (Figure 3(b)). 

(2) The chlorosome BChl *a *serves as the direct acceptor of exitation energy from BChl *c*, which was confirmed (a) by the presence of BChl *a* band in the fluorescence spectra of isolated chlorosomes both at room temperature and 77 K upon BChl *c* excitation (Figures 5(a) and 5(b)); (b) by the BChl *a* fluorescence excitation spectra that resembled the BChl *c* near-infrared absorption band both at room temperature and 77 K (Figure 4).

These data are in full agreement with our recent theoretical calculations that have shown the biological expedience of existence of an intermediate BChl *a *subantenna with its *Q*_*y*_ band being within the region of 790–800 nm [39]. Note that the shoulder at ~805 nm in the fluorescence spectrum of isolated chlorosomes (attributed to BChl *a* fluorescence, Figure 5(a)) is in a good agreement with this estimation of the position of BChl *a Q*_*y*_ transition that ensures the optimal coupling between chlorosome BChl *c* B750 subantenna and membrane BChl *a* B805-860 one [39]. 

(3) Upon alkaline treatment, only the 5.7 kDa CsmA protein was removed from the *Osc. trichoides* chlorosomes among five proteins detected by SDS-PAGE analysis (Figure 7(a)), concomitantly with the disappearance of BChl *a* fluorescence emission at 820 nm measured at 77 K (Figure 5(d)). The absorption bands of BChl *c*, the main light-harvesting pigment in *Osc. trichoides* chlorosomes, were practically not affected by alkaline treatment (Figure 3(a), dotted line). Note that ~6 kDa CsmA protein was found earlier in baseplates of *Chloroflexaceae *and* Chlorobiaceae* chlorosomes as a BChl *a*-binding protein.

Based on these results, we suggest that: (i) BChl *c* and BChl *a* are localized in two different neighboring substructures of *Osc. trichoides* chlorosomes, which is in excellent agreement with the data of electron microscopy (Figure 1); (ii) BChl *a *and CsmA 5.7 kDa are localized in one and the same substructure of *Osc. trichoides* chlorosomes, that is, out of the BChl *c* body and, therefore, in the baseplate of chlorosomes. 

So, we conclude that the intermediate-energy BChl *a* subantenna interfacing chlorosome B750 and membrane-bound B805-860 light-harvesting antennae is associated with 5,7 kDa CsmA protein and is located within the baseplate in *Osc. trichoides* chlorosomes. 

Thus, the presented results support our idea that the baseplate BChl *a* subantenna is a universal interface between the chlorosomal BChl *c* subantennae and the nearest BChl *a* ones in all three known families of green photosynthetic anoxygenic bacteria and represents a complex of BChl *a* with a ~6 kDa CsmA protein.

## Abbreviations

BChl: Bacteriochlorophyll
*Osc*: Oscillochloris
*Cfx*: Chloroflexus
*Chl*: Chlorobium
FWHM: Full width at half maximum
CBB: Coomassie brilliant blue R-250
SDS-PAGE: Sodium dodecyl sulfate polyacrylamide gel electrophoresis.
